# Supplementary material for: A New Structural Model of Apolipoprotein B100 Based on Computational Modeling and Cross Linking
Source: Int J Mol Sci. 2022 Sep 29;23(19):11480. doi: 10.3390/ijms231911480 (PMC9569473; doi:10.3390/ijms231911480)
Supplement: Supplementary file 1 [file ijms-23-11480-s001.zip › ijms-1917613-supplementary.pdf]

ID	Probability	P-value	Location	Sequence
A1	84.62	1.1e-06	1528-1551	---QGTNQITGRYEDGTLSLTSTSDLQ-s.........
A2	83.14	1.7e-07	1553-1578	--GIIKNTASLKYENYELTLKSDTNGKY..........
A3	78.53	5.4e-05	1579-1606	KNFATSNKMDMTFSKQNALLRSEYQADY..........
A4	75.93	5.0e-04	1607-1634	ESLRFFSLLSGSLNSHGLELNADILGTD..........
A5	81.46	8.0e-06	1635-1662	KINSGAHKATLRIGQDGISTSATTNLKC..........
A6	81.94	1.4e-06	1663-1690	SLLVLENELNAELGLSGASMKLTTNGRF..........
A7	76.18	3.6e-05	1691-1718	REHNAKFSLDGKAALTELSLGSAYQAMI..........
A8	81.28	4.5e-06	1719-1746	LGVDSKNIFNFKVSQEGLKLSNDMMGSY..........
A9	79.05	8.0e-05	1747-1774	AEMKFDHTNSLNIAGLSLDFSSKLDNIYss........
A10	76.64	1.1e-04	1777-1803	-DKFYKQTVNLQLQPYSLVTTLNSDLKY..........
A11	79.40	1.9e-06	1804-1831	NALDLTNNGKLRLEPLKLHVAGNLKGAY..........
A12	82.87	6.9e-07	1832-1859	QNNEIKHIYAISSAALSASYKADTVAKV..........
A13	80.53	6.1e-06	1860-1887	QGVEFSHRLNTDIAGLASAIDMSTNYNS..........
A14	80.99	4.3e-05	1888-1915	DSLHFSNVFRSVMAPFTMTIDAHTNGNGklalwgehtg
A15	73.06	1.1e-04	1926-1944	---QLYSKFLLKAEPLAFTFSH------..........

No. of repeats:15 P-value: 3.8E-14 Length:28

Figure S1. Subunit II domain 1 sequence repeats analysis. Fifteen rows show the structure aligned sequences of the repeats and are proceeded by the residue numbers. MPI Bioinformatics toolkit HHrepID was used for sequence analysis (https://toolkit.tuebingen.mpg.de/tools/hhrepid).

```
ID  Location    Probability   P-value
A1  2610-3126   100.00        1.9e-140
A2  3127-3673   100.00        3.3e-173
A3  3675-4059   100.00        2.9e-87

A1  2610-3126       --------------------------------------.-----------------------------------------
A2  3127-3673       NIPLTIPEMRLPYTIIITTPPLKDFSLWEKTGLKEFLK.TTKQSFDLSVKAQYKKNKHRHSITNPLAVLCEFISQSIKSFD
A3  3675-4059       ---------------IILP-VYDKSLWDFLKLDVTTSiGRRQHLRVSTAFVYTKNPNGYSFSIPVKVLADKFII--PG--

A1  2610-3126       --------------------------------------ATFQTPDFIVPLTDLRIPSVQINFKDLKNiKIPSRFSTPEFT
A2  3127-3673       RHFEKNRNNALDFVTKSYNETKIKFDKYKAEKSHDELPRTFQIPGYTVPVVNVEVSPFTIEMSAFGY.VFPKAVSMPSFS
A3  3675-4059       -------------------------------LKLNDLNSVLVMPTFHVPFTDLQVPSCKLDFREI--.QIYKKLRTSSFA

A1  2610-3126       I...........................LN-TFHIPSFTIdfvemkvkiirtidqmlnselqwpvpdiylrdlkvediPL
A2  3127-3673       I...........................LGSDVRVPSYTL......................................IL
A3  3675-4059       LnlptlpevkfpevdvltkysqpedsliPFFEITVPESQL......................................TV

A1  2610-3126       ARI.........................TLPDfrlpeiaipeFIIPTLnlndfqvPDLHIPEFQLPHISHTIEVPTFGKL
A2  3127-3673       PSL.........................ELPV..........LHVPRN.......LKLSLPDFKELCTISHIFIPAMGNI
A3  3675-4059       SQFtlpksvsdgiaaldlnavankiadfELPT..........IIVPEQ.......-TIEIPSIKF-SVPAGIVIPSFQAL

A1  2610-3126       YSILKIQSPLFTLDANADIGNGttsaneAG.IAASITAKGESKLEVLNFDFQANAQLSNPKinP.LALKESVKFSSKYLR
A2  3127-3673       TYDFSFKSSVITLNTNAELFNQ......SD.IVAHLLSSSSSVIDALQYKLEGTTRLTRKR..G.LKLATALSLSNKFVE
A3  3675-4059       TARFEVDSPVYNATWSASLKNK......ADyVETVLDSTCSSTVQFLEYELNVLGTHKIED..GtLASKTKGTFAHRDFS

A1  2610-3126       TEHGSEMLFFGNAIEGKSNTVASLHTEKNTLELSNGVIVKINNQLTLDSNTKYFHKLNIPKLDFSSQADLRNEIKTLLKA
A2  3127-3673       GSHNSTVSLTTKNMEVSVATTTKAQIPILRMNFKQELNGNTKSKPTVSSSMEFKYDFNSSMLYSTAKGAVDHKLSLESLT
A3  3675-4059       AEYEEDGKYEGLQ----------------------------------EWEGKAHLNIKSPAF---TDLHLRYQKDKKG--

A1  2610-3126       GHIAWTSSGKGSWKWAC......--PRFSDEGTHESQISFTIEGPLT.SFGLSNKINS---KHLRVNQNLVYESGSLN-F
A2  3127-3673       SYFSIESSTKGDVKGSV......LSREYSGTIASEANTYLNSKSTRS.SVKLQGTSKIDDIWNLEVKENFAGEATLQRIY
A3  3675-4059       ISTSAASPAVGTVGMDMdedddfSKWNFYYSPQSSPDKKLTIFKTELrVRESDEETQIKVNWE-----------------

A1  2610-3126       SKLEIQSQVDSQHVghsvltakgmalfgegkaeftgrhdaHlNGKVIGTLKNSLFFSAQPFEItastNNEGNLKVRFPLR
A2  3127-3673       SLWEHSTKNHLQLE..........................G.LFFTNGEHTSKATLELSPWQM....SALVQVHASQPSS
A3  3675-4059       --------------..........................-.---------------------....-------------

A1  2610-3126       LTGKIDFLNNYALFLSPSAQQASWQVSARFNQYKYNQNFSAGNNENIMEAHVGINGEANLDFL-.
A2  3127-3673       FHDFPDLGQEVALNANTKNQKIRWKNEVRIHSGSFQSQVELSNDQEKAHLDIAGSLEGHLRFLKn
A3  3675-4059       ----------------------------------------------------------------.

No. of repeats:3 P-value:3.3E-228 Length:527
```

Figure S2. Subunit IV (residues A2610-E4059) sequence repeats analysis. MPI Bioinformatics toolkit HHrepID was used for sequence analysis (https://toolkit.tuebingen.mpg.de/tools/hhrepid).

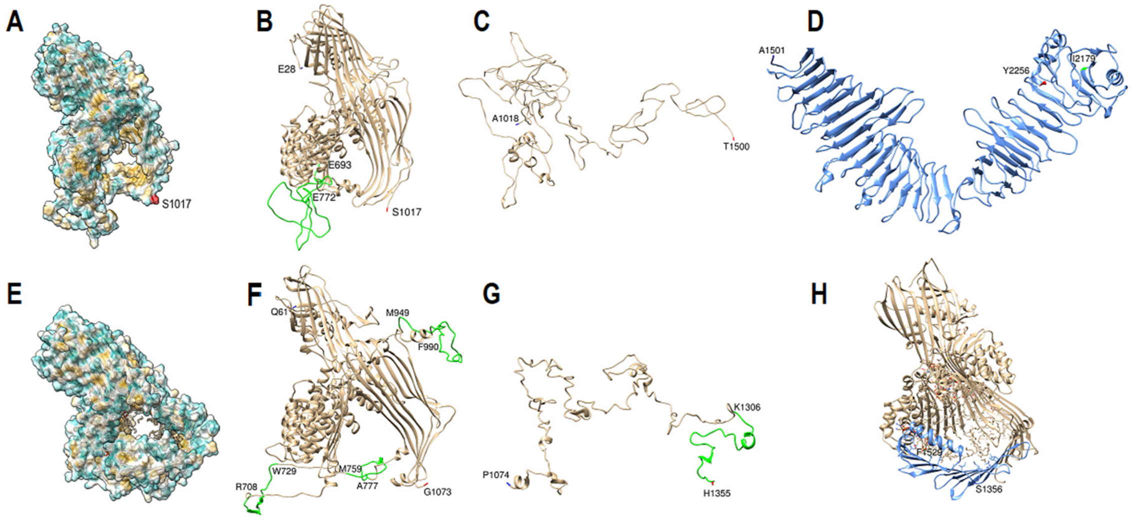

Figure S3. Human apoB and lamprey lipovitellin/vitellogenin secondary and tertiary structure comparison. (A) Surface representation of apoB residues E28-S1017 with the most hydrophobic (dark cyan) to white to least hydrophobic (dark goldenrod). S1017 in red indicates the C-terminal of subunit I. (B) Ribbon representation of apoB subunit I, which shows a large coil segment from E693-E772 in green. (C) ApoB subunit II coil, residues A1018-T1500. (D) ApoB subunit II domain 1 extended to subunit III domain 1, residues A1501-Y2256. Residue I2179 (green) indicates the C-terminal of apoB-48. (E) Surface representation of lipovitellin crystal structure with lipid molecules inside the cavity. (F) In a computational model of lipovitellin residues Q61-G1073, the three coils in green show the gaps in the crystal structure. (G) A computational model of vitellogenin residues P1074-H1355. Tan coil represents part of the vitellogenin sequence that is proteolytically cleaved, and the coil in green lacks electron density in X-ray crystallography. (H) Ribbon representation of lipovitellin crystal structure. (H) Ribbon representation of lipovitellin crystal structure with lipid molecules inside the cavity. The β-sheet in blue (S1356-F1529) that covers the base of the lipovitellin cavity is mainly made from β-strands and comparable to β-strands that form subunit II domain 1 in apoB. UCSF Chimera and ChimeraX were used for molecular graphics and analysis [1](https://www.rbvi.ucsf.edu/chimerax).

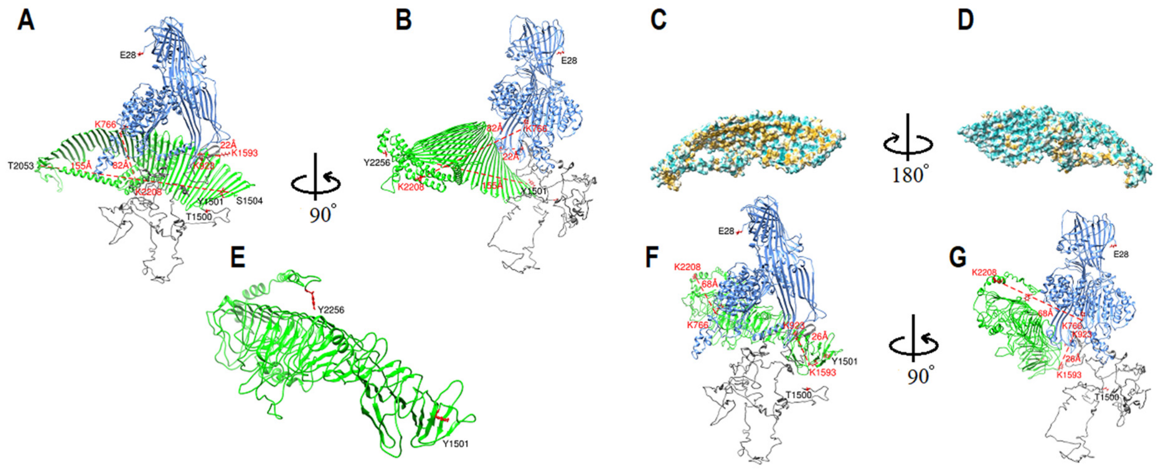

Figure S4. Human apoB-100 segment one (E28-T1500) and alternative segment two (Y1501-Y2256) match test by applying cross-links C (K766-K2208) and E (K923-K1593). (A-B) Ribbon representation of segments one and two indicate that the structure from Y1501-T2053 is folding in a β-sheet form and the distance between two residues is 155 Å. (C, D) Surface representation of segment two. The β-sheet was hydrophobic on one side (dark goldenrod) and hydrophilic (dark cyan) on the opposite side, but we were not able to orient the two segments according to the two cross-links. The distance between K766-K2208 was >26 Å (~82 Å). (E) Segment two's second model was a β-helix with a prism folding. (F, G) This model did not match segment one, and the distance between K766-K2208 was >26 Å (~68 Å). RoseTTAFold was used to generate the β-sheet model, and the β-helix model was made by I-TASSER [2–4].

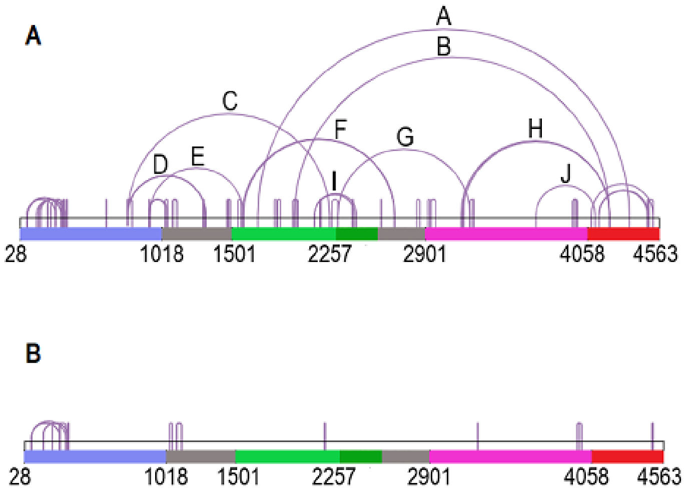

Figure S5. Comparison of DSSO cross-link maps on LDL versus VLDL. (A) DSSO cross-link map of linear apoB-100 sequence in LDL with inter-subunit cross-links are labeled from A-J. (B) DSSO cross-link map of VLDL lacks the inter-subunit and most of the short distance intra-subunit cross-links.

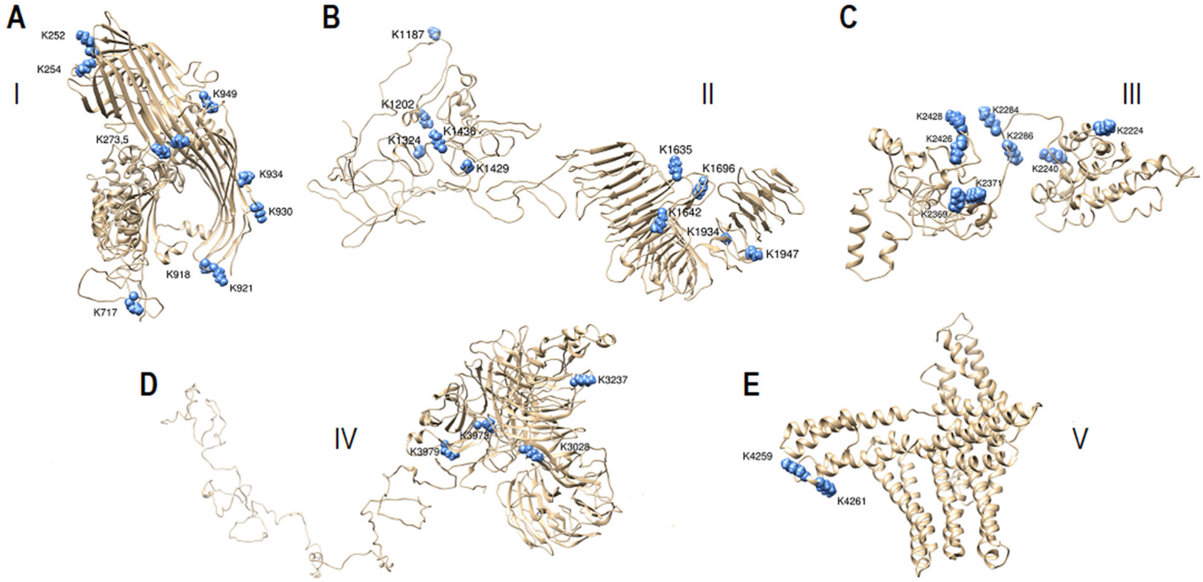

Figure S6. ApoB-100 structure with hydrolyzed DSSO cross-links. (A-E) Ribbon representation of apoB-100 five subunits with lysin spherical representation in blue indicates the location of hydrolyzed DSSO cross-links in LDL that determine hydrophilic areas.

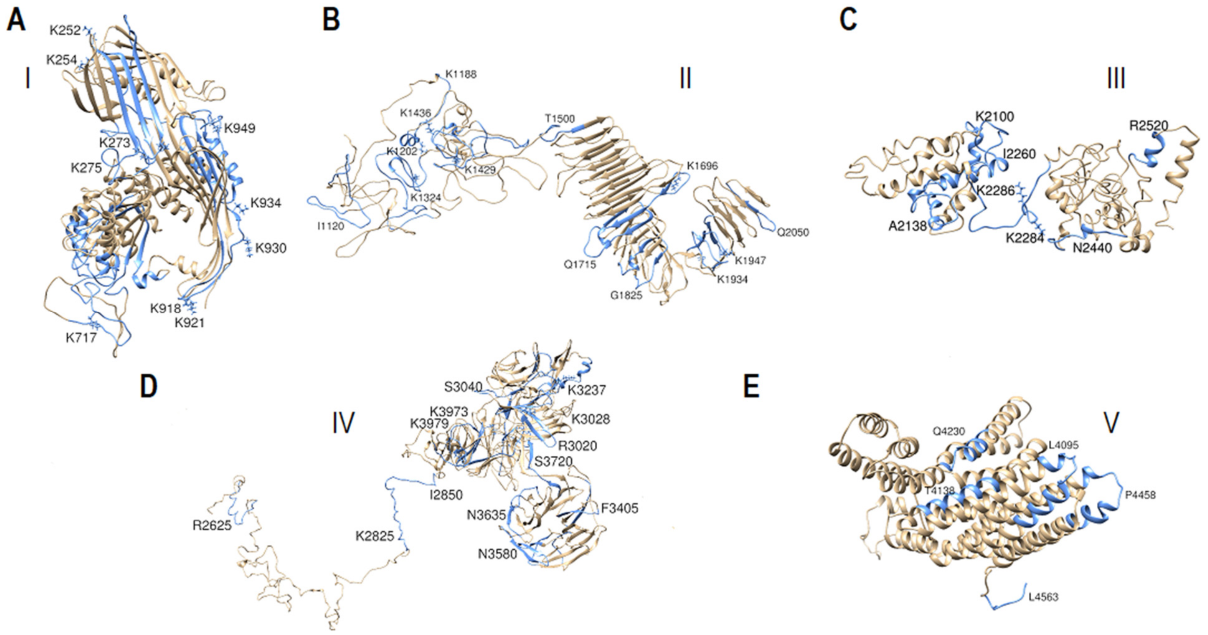

Figure S7. Painted ApoB-100 structure. (A-E) Ribbon representation of apoB-100 five subunits with painted regions in blue to determine hydrophilic areas. Lysin ball and stick representation in blue indicates the location of lysin residues those where painted and hydrolyzed. Most hydrolyzed DSSO cross-links (Fig. S6) are within the painted parts.

Table. S1. Human apoB-100 DSSO cross-links identified by nLC-MS/MS

Number	Peptide Sequence A	Position A	Peptide Sequence B	Position B	Distance (Cα-Cα) Å
1	[K]YTYNYEAESSSGVPGTADSR	51	LEDTP[K]INSR	293	6.3
2	YEL[K]LAIPEGK	132	[K]MGLAFESTK	305	8.5
3	YEL[K]LAIPEGK	132	FFGEGT[K]K	304	10.4
4	QVFLYPE[K]DEPTYILNIKR	147	FFGEGT[K]K	304	6.5
5	[K]GNVATEISTER	196	[K]HVAEAICKEQHLFLPFSYK	254	16.1
6	GMTRPLSTLISSSQSCQYTLDA[K]R	252	LEDTP[K]INSR	293	16
7	GMTRPLSTLISSSQSCQYTLDA[K]RK	252	[K]GNVATEISTER	196	16.1
8	GMTRPLSTLISSSQSCQYTLDA[K]R	252	F[K]HLR	47	10.2
9	YGMVAQVTQTLKLEDTP[K]INSR	293	F[K]HLRK	47	9.2
10	MGLAFEST[K]STSPPK	314	F[K]PIR	216	15.7
11	MGLAFEST[K]STSPPK	314	FFGEGT[K]K	304	19.7
12	STSPP[K]QAEAVLK	320	T[K]NSEEFAAAMSR	117	14.9
13	STSPPKQAEAVL[K]TLQELK	327	[K]LTISEQNIQR	334	11.1
14	NFVASHIANILNSEELDIQDL[K]K	612	LV[K]EALKESQLPTVMDFR	616	6.1
15	S[K]EVPEAR	768	LI[K]DLK	763	11.6
16	ILGEELGFASLHDLQLLG[K]LLLMGAR	797	LI[K]DLKSK	763	23.7
17	ILGEELGFASLHDLQLLG[K]LLLMGAR	797	LIKDL[K]SK	766	21.4
18	SGVQMNTNFFHESGLEAHVAL[K]AGK	918	L[K]FIIPSPK	923	9.5
19	ALVDTL[K]FVTQAEGAK	1034	QTEATMTF[K]YNR	1052	21.4
20	ALVDTL[K]FVTQAEGAK	1034	L[K]FIIPSPK	923	4.3
21	FVTQAEGA[K]QTEATMTFK	1043	AG[K]LK	921	10.9
22	VNDESTEG[K]TSYR	1087	[K]IKGVISIPR	1119	22.1
23	VNDESTEG[K]TSYR	1087	KI[K]GVISIPR	1121	16.4
24	IEIPLPFGG[K]SSR	1305	DL[K]MLETVR	1311	14.3
25	TPALHF[K]SVGFHLPSR	1324	LI[K]DLK	763	37.3
26	TPALHF[K]SVGFHLPSR	1324	DL[K]SK	766	42.5
27	EV[K]IDGQFR	1485	K[K]QHLFVK	1476	24.7
28	VSSFYA[K]GTYGLSCQRDPNTGR	1498	EV[K]IDGQFR	1485	25.6
29	YEDGTLSLTSTSDLQSGII[K]NTASLK	1556	SDTNG[K]YK	1577	14.6
30	NFATSN[K]MDMTFSKQNALLR	1586	V[K]IIR	2671	20.7
31	YKNFATSNKMDMTFS[K]QNALLR	1593	L[K]FIIPSPK	923	22.8
32	NFATSNKMDMTFS[K]QNALLR	1593	V[K]IIR	2671	18.6
33	FREHNA[K]FSLDGK	1696	LL[K]ENLCLNLHK	4349	22.7
34	LHVAGNL[K]GAYQNNEIK	1828	YNALDLTNNG[K]LR	1813	22.8
35	GAYQNNEI[K]HIYAISSAALSASYK	1837	ADTVA[K]VQGVEFSHR	1858	8.2
36	AEPLAFTFSHDY[K]GSTSHHLVSR	1947	VSALLTPAEQTGTW[K]LK	1982	23.3
37	AEPLAFTFSHDY[K]GSTSHHLVSR	1947	L[K]TQFNNNEYSQDLDAYNTK	1984	21.4
38	SISAALEH[K]VSALLTPAEQTGTWK	1967	AEPLAFTFSHDY[K]GSTSHHLVS R	1947	16.1

39	NL[K]HINIDQFVR	2100	LVGFIDDAV[K]K	2402	33.9
40	NL[K]HINIDQFVR	2100	I[K]DYFEK	2387	36.3
41	QVSHA[K]EK	2139	LTALT[K]K	2147	14.8
42	IAIANIIDEIIE[K]LK	2208	LIKDL[K]SK	766	22.9
43	VNLV[K]TIHDLHLFIENIDFNK	2224	LQQL[K]R	2270	18.4
44	ETIQ[K]LSNVLQQVK	2376	LVELAHQY[K]LK	2369	9.7
45	NLTDFAEQYSIQDWA[K]R	2575	M[K]ALVEQGFTVPEIK	2578	N/A
46	TEHGSEMLFFGNAIEG[K]SNTVASLHTE K	2853	ESV[K]FSSK	2829	28
47	AGHIAWTSSG[K]GSWK	2926	NEI[K]TLLK	2911	34.2
48	GSW[K]WACPR	2930	INS[K]HLR	2966	17.1
49	LPYTIITTPPL[K]DFSLWEK	3148	FQFPG[K]PGIYTR	4207	18.5
50	LPYTIITTPPLKDFSLWEKTGL[K]EFLK	3159	FQFPG[K]PGIYTR	4207	19.2
51	TT[K]QSFDLSVK	3166	TGL[K]EFLK	3159	10.9
52	SFDRHFE[K]NR	3210	LQQL[K]R	2270	19.8
53	I[K]FDKYK	3229	YKAE[K]SHDELPR	3237	22.8
54	IKFD[K]YK	3232	AE[K]SHDELPR	3237	13
55	AE[K]SHDELPR	3237	HFE[K]NR	3210	25.1
56	NIILPVYD[K]SLWDFLK	3682	EVSS[K]LR	4103	25.1
57	DFSAEYEEDG[K]YEGLQEWEGKAHLNI K	3963	T[K]GTFAHR	3946	11.1
58	DFSAEYEEDGKYEGLQEWEG[K]AHLNI K	3973	T[K]GTFAHR	3946	26.9
59	AHLNI[K]SPAFTDLHLR	3979	T[K]GTFAHR	3946	12
60	DNVP[K]ATGVLYDYVNK	4076	FIAES[K]R	4518	17.2
61	FQ[K]AASGTTGTYQEWKDK	4132	SQAIAT[K]K	4485	18.2
62	FQFPG[K]PGIYTR	4207	[K]SISAALEHK	1958	21.1
63	IAELSATAQEII[K]SQAIATK	4478	[K]IISDYHQQFR	4486	12.3
64	IAELSATAQEII[K]SQAIATKK	4478	FQ[K]AASGTTGTYQEWKDK	4132	24.8
65	SQAIAT[K]K	4485	FIAES[K]R	4518	28

A total number of 65 unique cross-links were identified by mass spectrometry. The distance between Cα-Cα of lysine residues was measured except for cross-link n:45, which was located at the beginning of the subunit IV gap. 56 cross-links were within the 26 Å threshold, and 8 cross-links were beyond the 26 Å limit (red). At least one lysine residue of cross-links n:25, n:26, and n:46 was located within the coil region.

References

(1) Pettersen, E. F.; Goddard, T. D.; Huang, C. C.; Couch, G. S.; Greenblatt, D. M.; Meng, E. C.; Ferrin, T. E. UCSF Chimera—A Visualization System for Exploratory Research and Analysis. *J Comput Chem* 2004, *25* (13), 1605–1612. https://doi.org/10.1002/jcc.20084.

(2) Zhang, C.; Freddolino, P. L.; Zhang, Y. COFACTOR: Improved Protein Function Prediction by Combining Structure, Sequence and Protein–Protein Interaction Information. *Nucleic Acids Res* 2017, *45* (Web Server issue), W291–W299. https://doi.org/10.1093/nar/gkx366.

(3) Yang, J.; Zhang, Y. I-TASSER Server: New Development for Protein Structure and Function Predictions. *Nucleic Acids Res* 2015, *43* (W1), W174–W181. https://doi.org/10.1093/nar/gkv342.

(4) Baek, M.; DiMaio, F.; Anishchenko, I.; Dauparas, J.; Ovchinnikov, S.; Lee, G.; Wang, J.; Cong, Q.; Kinch, L. N.; Schaeffer, D. R.; Millán, C.; Park, H.; Adams, C.; Glassman, C. R.; DeGiovanni, A.; Pereira, J. H.; Rodrigues, A. V.; Dijk, A. A. van; Ebrecht, A. C.; Opperman, D. J.; Sagmeister, T.; Buhlheller, C.; Pavkov-Keller, T.; Rathinaswamy, M. K.; Dalwadi, U.; Yip, C. K.; Burke, J. E.; Garcia, C. K.; Grishin, N. V.; Adams, P. D.; Read, R. J.; Baker, D. Accurate Prediction of Protein Structures and Interactions Using a Three-Track Neural Network. *Science* 2021, eabj8754. https://doi.org/10.1126/science.abj8754.
